# Evaluation of dental enamel microproperties after bleaching with 35% hydrogen peroxide and different light sources: An *in vitro* study

**DOI:** 10.4317/jced.58440

**Published:** 2021-10-01

**Authors:** Alieny-Cristina-Duarte Ferreira, Ana-Luzia-Araújo Batista, José-de Alencar-Fernandes Neto, Thamyres-Maria-Silva Simões, Maria-das Graças-Barbosa da Silva, Daiane-Domingos de Barros, Josefa-Simere-dos Santos-Barros Catão, Tiago-Almeida de Oliveira, Maria-Helena-Chaves-de Vasconcelos Catão

**Affiliations:** 1 Master’s Student of the Graduate Postgraduate Program in Dentistry, State University of Paraiba, R. Baraúnas, 351, Bodocongó, Campina Grande, PB, 58429-500, Brazil; 2 PhD student of the Postgraduate Program in Dentistry, State University of Paraiba, R. Baraúnas, 351, Bodocongó, Campina Grande, PB, 58429-500, Brazil; 3 Dentistry Undergraduates from the State University of Paraiba, R. Baraúnas, 351, Bodocongó, Campina Grande, PB, 58429-500, Brazil; 4 Dentistry undergraduates from the Integrated Faculty of Patos, R. Floriano Peixoto, 3333, Santa Rosa, Campina Grande, PB, 58416-440, Brazil; 5 Teacher of the Department of Statistics, State University of Paraiba, R. Baraúnas, 351, Bodocongó, Campina Grande, PB, 58429-500, Brazil; 6 Teacher of the Postgraduate Program in Dentistry, State University of Paraiba, R. Baraúnas, 351, Bodocongó, Campina Grande, PB, 58429-500, Brazil

## Abstract

**Background:**

To evaluate the tooth enamel surface morphology after the action of 35% hydrogen peroxide with and without LED activation.

**Material and Methods:**

70 bovine incisors with an enamel surface of 4x4x3 mm were used, prepared for reading superficial microhardness and roughness. Specimens were randomly distributed and divided into 7 experimental groups (n = 10); G1 = artificial saliva; G2 = 35% HP - 2 sessions (3x15´); G3 = Phosphoric Acid + 35% HP - 3 sessions (3x15´); G4 = 35% HP - 2 sessions (3x15´) + blue LED; G5 = 35% HP - 2 sessions (3x15´) + green LED; G6 = 35% HP - 2 sessions (3x20´) + violet LED; G7 = Violet LED - 2 sessions (3x20´). The results were analyzed by the Anova, Wilcoxon, Dunnett and Tukey tests (α = 0.05).

**Results:**

The G4 group showed a greater change in microhardness. Regarding roughness, the biggest mean difference between groups occurred in G2, G5 and G7. Optical microscopy showed a smooth enamel surface in groups G2, G5 and G7.

**Conclusions:**

Changes in the enamel surface were observed in relation to microhardness, but without significant changes in roughness, where the LED (green and violet) resulted in a smooth surface.

** Key words:**Tooth whitening, superficial morphology, light, photoradiatio.

## Introduction

Tooth whitening occurs when there is intimate contact between mineralized dental tissues and the bleaching substance, which has active agents capable of promoting the removal of pigments intrinsic of the dental structure formed by long and complex carbon chains (macromolecules), which are difficult to be eliminated by the dental structure. Through oxidation-reduction reactions, oxygen interacts with pigmented molecules, being able to break them into smaller and less pigmented molecules and then be eliminated (1).

There are two main tooth whitening techniques, the at-home technique and the in-office technique. The in-office whitening treatment is carried out using higher concentrations of peroxides, such as 35% hydrogen peroxide, and most of these peroxides are activated with the help of a light energy source, such as light-emitting diodes (LED), providing satisfactory and fast results. LED applications are generally performed in two to four sessions at seven-day intervals (2).

Light-emitting diode (LED) is a device that produces heat and can promote tooth whitening without significant risks. These devices have been associated with products with sensitive photoactivators that absorb energy and activate hydrogen peroxide. In addition, they require little energy to generate light, thus being a simple and economical alternative to lasers (3).

LEDs have photochemical (chemical interaction of the light source with bleaching agents) and photothermal effect (slight increase in temperature). It is noteworthy that many LED devices have been introduced in the dental market for the photoactivation of composites; however, unlike devices intended for use in in-office dental bleaching, those used in restorative procedures have high power intensity and generate excessive heat if used in the time required for tooth whitening (4).

The emission spectrum of LED systems used for tooth whitening is located within the blue spectrum, and therefore does not extend to the infrared spectrum, such as tungsten quartz and plasma arc lamps. In tooth whitening, LED systems are able to promote greater comfort to patients (5).

LED light (violet light) presents emission of photons (energy packages) that propagate at shorter wavelength and higher vibrational frequency in relation to blue light, which gives it physical characteristic of lower penetrability in the dental tissue and greater delivery of energy on surfaces, where this physical property of violet light is advantageous, reaching surface molecules that pigment the teeth with greater energy, breaking bonds present in molecular chains that form these pigments (6).

Literature shows that, after whitening, subclinical changes in the superficial micromorphology of dental tissues can occur, leading to greater sensitivity, increased porosity and surface roughness, in addition to decreased microhardness, with emphasis on enamel. Data referring to changes in dental tissues are conflicting due to the wide variety of methodologies used, as well as to the diversity of bleaching agents, application time, concentrations, commercial brands and association with light sources (7).

Therefore, due to the great controversy observed in literature regarding the effects of bleaching materials on dental surfaces, the aim of the present study was to evaluate the dental enamel morphology after the action of bleaching agents by measuring the roughness parameters, the microhardness values and Optical Microscopy (OM) of the bovine enamel surface submitted to bleaching with 35% hydrogen peroxide with and without light activation.

## Material and Methods

The present *in vitro* experimental study was composed of a sample of 70 bovine incisors that were sectioned at the cementoenamel junction , separating the crown from roots, perpendicularly in relation to the long axis of the tooth with the aid of a drill (Carbide Fg 702 19mm Prima Angelus, Londrina / PR, Brazil). Then, longitudinal and cross sections were made in the crown with precision cutter (Isomet 1000-Buehler Ltda, Illinois, USA), obtaining 70 enamel and dentin specimens (4x4x3 mm), which were randomly divided into seven experimental groups (n = 10).

**Figure 1:**
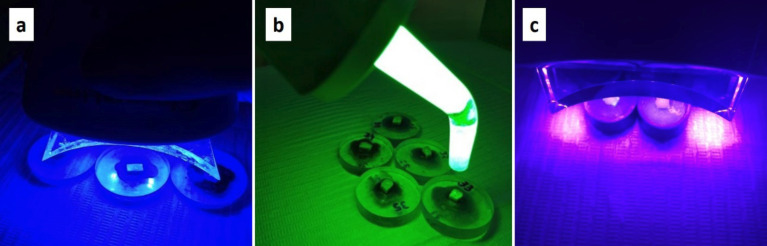
a) Application of 35% HP whitening gel with blue LED light (λ 470nm) in G4. b) Application of 35% HP whitening gel with green LED light (λ 530nm) in G5. c) Application of the violet LED λ 400nm ± 10nm in groups 6 and 7.

Specimens were randomly divided into 7 groups (n = 10): G1 control (artificial saliva), G2 - 35% hydrogen peroxide (Lase Peroxide, DMC Equipamentos, São Carlos - SP, Brazil) with two sessions of three fifteen-minute applications in each session; G3 - 37% phosphoric acid for 30 seconds, then two sessions of three fifteen-minute applications in each session with 35% hydrogen peroxide; G4 - 35% hydrogen peroxide in two sessions of three fifteen-minute applications in each session plus activation of blue LED light (λ 470nm, BrigHTMaXEvolution - MMOptics, São Carlos, SP, Brazil) in the last three minutes of each application (Fig. 1a); G5 - 35% hydrogen peroxide in two sessions of three fifteen-minute applications in each session plus green LED light activation (λ 530nm, Kondortech, São Carlos, SP, Brazil) in the last three minutes of each application (Fig. 1b); G6 - 35% hydrogen peroxide in two sessions of three twenty-minute applications in each session plus simultaneous violet LED light activation (λ 400nm ± 10nm, Bright Max Whitening-MMO, São Carlos, SP) (Fig. 1c); G7 - violet LED activation (λ 400nm ± 10nm), for twenty minutes in two sessions of three applications, with interval of 7 days between each session.

All specimens were submitted to the surface microhardness test before and after bleaching. Microhardness measurements were performed in Knoop hardness testing machine (HMV-2000 Shimadzu, Tokyo, Japan), with three indentations, one performed at the center of the fragment, one above and one under the first one. The test was carried out under load of 100 grams, with penetration time of ten seconds. Regarding indentation of extremities, the distance of 1 mm from the margin of the specimen was respected in order to obtain the result without weakening the material. Microhardness tests were read with Vickers diamond tip, which produces a square-shaped indentation.

For the enamel surface analysis, Optical Microscopy (OM) was used, where random draw was carried out within each experimental group with n = 5, using digital microscope (HIROX ® model KH-7700, Tokyo, Japan, obtaining optical photomicrographs in two different regions of the buccal enamel surfaces with 140x magnification. The average roughness (Ra) was analyzed after the end of the treatment of each experimental group using the Gwyddion Software version 2.4 for windows. Through this software, 70 photomicrographs captured in the OM were analyzed. Microhardness data were submitted to the Wilcoxon test; roughness was analyzed using ANOVA with F test, Dunnett and Tukey test, all at 5% significance level.

## Results

The descriptive analysis of data obtained at the different microhardness assessment times resulted in average initial and final values for experimental and control groups. Control group and group 6 showed increase in microhardness, while group 3 and 4 significantly reduced enamel microhardness, as described in Table 1.

**Table 1:**
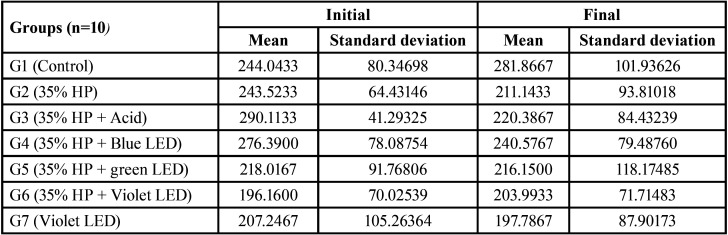
Microhardness result of the initial and final enamel.

The results of microhardness measurements are shown in Table 2, according to the average scores for each group individually, where according to the Wilcoxon Signed Classifications test, the group that showed the greatest significance for microhardness alteration was G4 group (0.028) followed by G3 group (0.059).

**Table 2:**
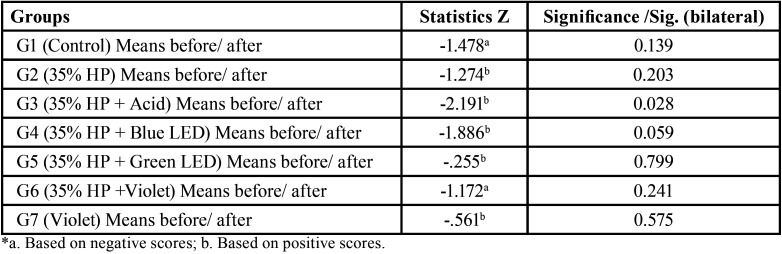
Wilcoxon microhardness test statistics.

Regarding the average roughness (Ra) after treatments, it was observed that according to Table 3, when comparing control group to groups that performed the bleaching techniques, groups that obtained averages above the group control were G2, G5 and G7.

**Table 3:**
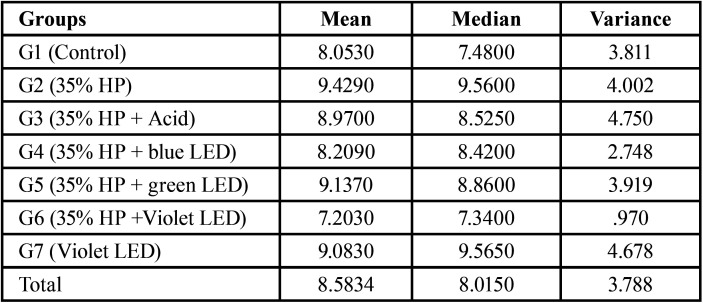
Average and Median Roughness values, Average (Ra) after treatments.

To compare the control group with the other groups, the Snedecor F test was used (*p* value> 0.05), which is not significant, and the Dunnett test, which treats one group as a control and compares all other groups with it, as shown in Table 4; the highest mean difference between groups with and without treatment occurred in G2, G5 and G7, but without any significant difference.

**Table 4:**
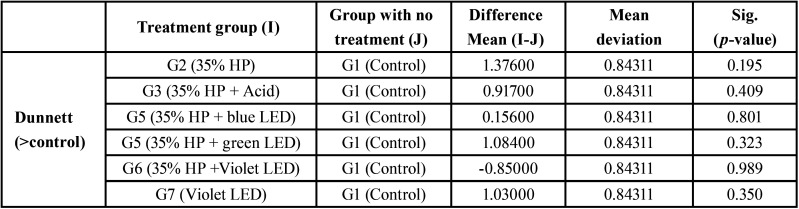
Comparison between treatment groups and control group.

**Figure 2:**
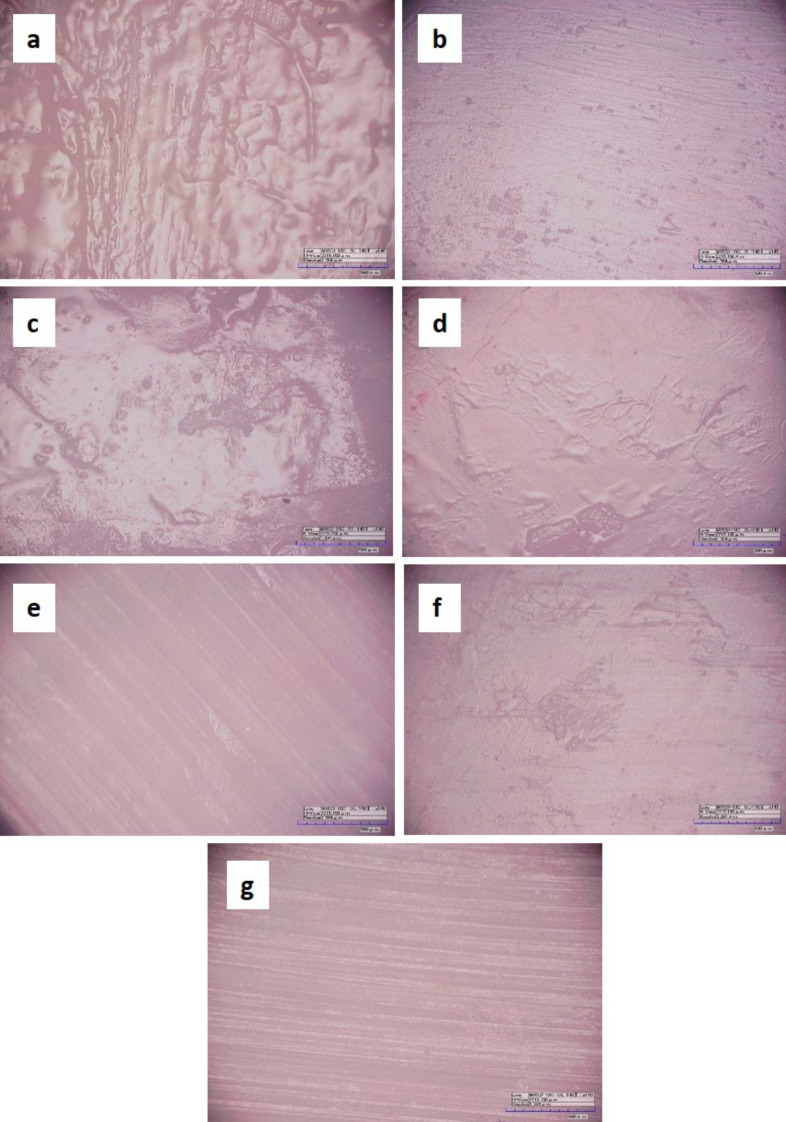
a) OM of G1 surfaces (artificial saliva). b) OM of G2 surfaces (35% HP). c) OM of G3 surfaces (Phosphoric Acid + 35% HP). d) OM of G4 surfaces (35% HP + blue LED). e) OM of G5 surfaces (35% HP + green LED). f) OM of G6 surfaces (35% HP + violet LED). g) OM of G7 surfaces (violet LED).

OM performed after bleaching treatment of specimens showed that for all types of bleaching, alterations were found, with microscopic difference in the photomicrographs of enamel surfaces when compared to control group, with greater differences in the following groups: G2 (35% HP); G5 (35% HP + Green LED) and G7 (Violet LED) (Fig. 2).

## Discussion

In recent years, many studies have tried to understand the effectiveness of whitening treatments, testing and evaluating different products and techniques according to a defined clinical protocol. This study was developed to evaluate the morphological alterations of the dental enamel using 35% hydrogen peroxide and LED in the treatment.

Artificial saliva was also used in this study, as a control group for clinical simulation purposes, avoiding specimen demineralization, which could interfere with results. The use of peroxide-based bleaching agents and the development of techniques that produce a more quick whitening effect have been discussed in literature, as they can cause tooth sensitivity and changes in enamel morphology (8).

The microhardness test is a simple technique often used to determine the mechanical enamel and dentin properties after the whitening process. In the present study, bleaching with 35% peroxide in association with light source resulted in significant decrease in microhardness. Reduction was approximately 10% for the technique and indicated a possible change in the enamel surface morphology. The results of this study corroborate those of Ghanbarzadeh *et al*., (9) who reported that the enamel microhardness significantly decreased after whitening performed on forty bovine incisors with 40% hydrogen peroxide and irradiated with 810 nm gallium-aluminum-gallium (GaAlAs) diode laser (CW, 2W) performed for three sessions every seven days, for fifteen days.

In the study by Pimenta-Dutra *et al*. (10) changes in the enamel surface of ninety bovine teeth through SEM after bleaching with exogenous agents: 10% carbamide peroxide, 16% carbamide peroxide and 35% hydrogen peroxide activated by LED, showed changes in the enamel surfaces of specimens that received treatment with 35% hydrogen peroxide and LED activation, corroborating results of this study when comparing the average roughness values of experimental groups treated with 35% hydrogen peroxide and LED light source, with no statistical significance between experimental groups.

In this study, the experimental group that received the 35% hydrogen peroxide bleaching treatment in association with violet light source also showed increase in microhardness after treatment, which can be clinically explained by Santos, Bussadori, Pinto (11) , who described a protocol for the first randomized controlled clinical trial to compare the effects of the two methods (violet LED and 35% carbamide peroxide), showing that violet LED is as effective as the standard tooth whitening procedure used in most studies. In addition, this treatment has the advantage of not causing tooth sensitivity or damage to gingival tissue.

In the study by Zanin *et al*. (4) using the combination of LED and laser irradiance in teeth submitted to whitening, the scanning electron microscopy analysis showed smooth surface with well-formed hydroxyapatite prisms, with removal of the organic matrix without alteration of the mineral structure. It was also observed that the selective radiation of the LED system can reduce the whitening time without significant enamel surface modifications. However, in disagreement with results found in this research, the present study found through optical microscopy differences in the enamel surface of groups that had LED activation (green and violet) compared to the control group.

Regarding roughness, changes in physical properties can occur due to the effects of hydrogen peroxide diffusion and the acidic pH of bleaching products. This process occurs irregularly, inducing the reorganization of enamel prisms that can increase roughness, as observed in the present study, only in groups bleached with 35% peroxide and in groups with the association of LED (green and violet), although with no statistically significant significance between experimental groups. When determining the enamel surface roughness in twenty-seven bovine incisors submitted to bleaching with and without laser activation, Xavier *et al*. (7) found that the use of laser in bleaching did not increase the superficial enamel roughness of bovine teeth.

However, the results of the present study are in disagreement with those obtained in the in situ study by Silva *et al*., (12) who evaluated the influence of 10% carbamide peroxide on the enamel physical characteristics of eighty-four bovine enamel and dentin blocks divided into seven groups, fixed in intraoral palatal devices, six of which submitted to treatments and one control group, and observed increase in roughness in all bleaching groups when compared to the control group. Therefore, it should also be considered that, although many *in vitro* studies have found changes in the surface structure of the dental enamel, the results should be extrapolated to clinical practice with caution, since in the oral cavity, teeth are submitted to the remineralizing action of saliva and the presence of fluorides.

## Conclusions

Based on the methodology and results obtained, it could be concluded that:

● Group (35% HP activated by blue LED) was able to promote greater changes in surface microhardness of the dental enamel, compared to the other groups that used whitening with light source activation;

● Group (35% HP activated by violet LED) showed increase in microhardness and decrease in roughness;

● All techniques used with or without LED light activation did not promote significant changes in enamel roughness;

● Regarding surface morphology, the use of LED (green and violet) caused greater change in the enamel surface with exposure of smooth surface, a characteristic not found when using 35% HP alone or activated by blue LED.
